# Prognostic Significance of Autophagy-Relevant Gene Markers in Colorectal Cancer

**DOI:** 10.3389/fonc.2021.566539

**Published:** 2021-04-15

**Authors:** Qinglian He, Ziqi Li, Jinbao Yin, Yuling Li, Yuting Yin, Xue Lei, Wei Zhu

**Affiliations:** ^1^ Department of Pathology, Guangdong Medical University, Dongguan, China; ^2^ Department of Pathology, Dongguan People’s Hospital, Southern Medical University, Dongguan, China

**Keywords:** colorectal cancer, autophagy, prognostic model, splicing, markers

## Abstract

**Background:**

Colorectal cancer (CRC) is a common malignant solid tumor with an extremely low survival rate after relapse. Previous investigations have shown that autophagy possesses a crucial function in tumors. However, there is no consensus on the value of autophagy-associated genes in predicting the prognosis of CRC patients. This work screens autophagy-related markers and signaling pathways that may participate in the development of CRC, and establishes a prognostic model of CRC based on autophagy-associated genes.

**Methods:**

Gene transcripts from the TCGA database and autophagy-associated gene data from the GeneCards database were used to obtain expression levels of autophagy-associated genes, followed by Wilcox tests to screen for autophagy-related differentially expressed genes. Then, 11 key autophagy-associated genes were identified through univariate and multivariate Cox proportional hazard regression analysis and used to establish prognostic models. Additionally, immunohistochemical and CRC cell line data were used to evaluate the results of our three autophagy-associated genes EPHB2, NOL3, and SNAI1 in TCGA. Based on the multivariate Cox analysis, risk scores were calculated and used to classify samples into high-risk and low-risk groups. Kaplan-Meier survival analysis, risk profiling, and independent prognosis analysis were carried out. Receiver operating characteristic analysis was performed to estimate the specificity and sensitivity of the prognostic model. Finally, GSEA, GO, and KEGG analysis were performed to identify the relevant signaling pathways.

**Results:**

A total of 301 autophagy-related genes were differentially expressed in CRC. The areas under the 1-year, 3-year, and 5-year receiver operating characteristic curves of the autophagy-based prognostic model for CRC were 0.764, 0.751, and 0.729, respectively. GSEA analysis of the model showed significant enrichment in several tumor-relevant pathways and cellular protective biological processes. The expression of EPHB2, IL-13, MAP2, RPN2, and TRAF5 was correlated with microsatellite instability (MSI), while the expression of IL-13, RPN2, and TRAF5 was related to tumor mutation burden (TMB). GO analysis showed that the 11 target autophagy genes were chiefly enriched in mRNA processing, RNA splicing, and regulation of the mRNA metabolic process. KEGG analysis showed enrichment mainly in spliceosomes. We constructed a prognostic risk assessment model based on 11 autophagy-related genes in CRC.

**Conclusion:**

A prognostic risk assessment model based on 11 autophagy-associated genes was constructed in CRC. The new model suggests directions and ideas for evaluating prognosis and provides guidance to choose better treatment strategies for CRC.

## Introduction

Colorectal cancer (CRC) is a prevalent disease worldwide (1). Even with improvements in living standards and changes in dietary structure, the morbidity and fatality of CRC have remained high in recent years (2). At present, the prognosis of CRC patients is primarily assessed using the tumor-node-metastasis (TNM) staging system. Generally, the earlier the stage it occurs, the better the prognosis is. Autophagy is a lysosome-dependent degradation pathway characterized by cytoplasmic vacuolation (3). It can degrade damaged structures in the cytoplasm and produce small organic molecules, and other substances for protein compound and energy metabolism, enabling cells to adapt to hypoxia and starvation (4). The process of autophagy is modulated by multifarious complex signaling molecules (5). Failure of this regulatory mechanism is closely related to tumor development, neurodegenerative diseases, and aging (6–8). Current experimental data demonstrate that autophagy is related to CRC (9). Raptor and autophagy-related 5 (ATG5), which are autophagy-related genes, contribute to CRC metastasis and drug resistance by regulating autophagy (10, 11), suggesting that autophagy may be significant in treatment and prognosis prediction for CRC. Although many investigations have explored the pathogenesis of CRC, further studies are needed to fully elucidate its detailed molecular mechanisms.

In the present study, autophagy-associated genes differentially expressed in CRC were screened using a bioinformatics approach. In addition, a simple prognostic model of autophagy-associated genes related to the prognosis of CRC patients was constructed based on Cox analysis, in order to obtain evidence for the application of these genes in prognosis prediction and clinical treatment of CRC patients.

## Materials and Methods

### Data Download and Identification of Differentially Expressed Genes

Raw CRC transcript data, clinical patient data, and the metadata for all DNA whole-exome BAM files were collected from The Cancer Genome Atlas (TCGA) database (https://portal.gdc.cancer.gov/; released before October 27, 2019) (12), and data for 1,526 autophagy-associated genes with a relevance score of >1.5 were obtained from the GeneCard database (https://www.genecards.org; released before February 19, 2020) (13). As 15 genes, including MARCHF7, CCN2, H2AX, and SARS1, were not found in the TCGA CRC sample, only 1,511 autophagy-associated genes were obtained. TCGA CRC mutation data (VarScan2 Variant Aggregation and Masking) were downloaded from UCSC (https://xenabrowser.net/datapages/; released before March 30, 2020) (14). Sample expression values from the above data were collated using perl (version 10.0.18363.1256), and the ENSEMBL gene ID was converted into a gene name. The Wilcox test was performed on the autophagy-associated genes from CRC tissues and normal tissues using the R software (version 3.6.1) “limmar” package, and the differentially expressed genes (DEGs) were defined by the absolute values of log (fold change) >1 and FDR (false discovery rate) <0.05.

### Prognostic Model Construction

DEGs were subjected to univariate Cox analysis using the “survival” R package. Autophagy-associated genes related with CRC prognosis were identified, and multivariate Cox analysis was carried out to construct a prognostic model according to the best Aike information criterion. 

### Assessment of the Accuracy of the Prognostic Model

By combining the expression of autophagy-associated genes and the coefficients obtained by multivariate Cox regression analysis, a final formula for calculating risk scores was listed next: risk scores = SLCO1A2exp × 1.24168405018345 + RAB6Bexp × 0.425074259002984 + SNAI1exp × 0.480196885198325 + NOL3exp × 0.473830469554988 − ULK4exp × 1.04014423940406 − EPHB2exp × 0.295212904754073 + TRAF5exp × 0.379124034189421 − PPARGC1Aexp × 0.516127561999247 + MAP2exp × 0.742235003720545 − RPN2exp × 0.557629664887039 − IL13exp × 6.47343548405588. According to the above formula, we calculated the prognostic risk value for each sample in the TCGA data (the median value of the risk score was the standard for defining the high-risk and low-risk groups). Then, the “survival” and “survminer” R packages were used for survival analysis and independent prognosis analysis using the risk score, as well as for visualization of the results. We also performed Kaplan-Meier (KM) survival analysis of 11 autophagy genes in the model. Receiver operating characteristic (ROC) analysis was conducted using the “survivalROC” R package. Finally, the concordance index (C-index) of the autophagy-related prognostic model was calculated. The values of C-index and AUC have low accuracy when they are from 0.50 to 0.70, moderate accuracy when they are from 0.71 to 0.90, and high accuracy when they are greater than 0.90. 

### Evaluation of EPHB2, NOL3, and SNAI1

For the evaluation of protein level, IHC samples of CRC were downloaded from EPHB2, NOL3, and SNAI1 in the human protein atlas (HPA) database (https://www.proteinatlas.org/) (15) and the integrated optical density (IOD) was analyzed. The IOD of IHC was analyzed by Image-Pro Plus 6.0 software and GraphPad software 8 (statistical significance between groups was examined with an independent-samples t-test). For the evaluation of cell level, the verification of EPHB2, NOL3, and SNAI1 was performed using the Cancer Cell Line Encyclopedia (CCLE) database (https://portals.broadinstitute.org/ccle/about) (16), and GraphPad software 8 was used to visualize the mRNA expression of these genes in nine common CRC cell lines (CACO2, HCT116, HT29, LOVO, RKO, SW1116, SW48, SW480, and SW620) obtained from the CCLE database. When the *p* value is less than 0.05, the data are considered to be statistically significant. 

### Identification of Related Signaling Pathways

To identify potential signaling pathways related to prognosis in CRC, we divided CRC patients into high- and low-risk groups for Gene Set Enrichment (GSEA) analysis using the prognostic model. To determine the biological functions and signaling pathways related to the genes in the prognostic model, the target genes of 11 autophagy genes in the model were obtained using the online RNA prediction platform ENCORI (encyclopedia of RNA interactions, http://starbase.sysu.edu.cn/rbpClipRNA.php?source=mRNA; released before February 19, 2020) (17), and Gene Ontology (GO) and Kyoto Encyclopedia of Genes and Genomes (KEGG) analyses were carried out. 

### The Relationship Between the Expression of 11 Autophagy-Associated Genes and MSI and TMB

According to the previous steps (18), the algorithm MANTIS was used to calculate the MSI score of each sample of CRC, and then R was used to analyze the correlation between these autophagy-related genes and MSI (Spearman), and draw the radar map of the correlation between these genes and MSI. Perl was used to collate the TCGA CRC mutation data obtained from UCSC and calculate the TMB data of each sample. The TMB data of each sample were collated and calculated by perl, and the TMB was calculated as the total number of somatic mutations (including non-synonymous point mutations, insertions, and deletions in the coding region of exons)/the size of the target region, in units of mutations/Mb. Then R was used to analyze the correlation between these autophagy-related genes and TMB (Spearman). Finally, the radar map of the correlation between these genes and TMB was drawn.

## Results

### Differential Expression of Autophagy-Associated Genes

**Figure 1:**
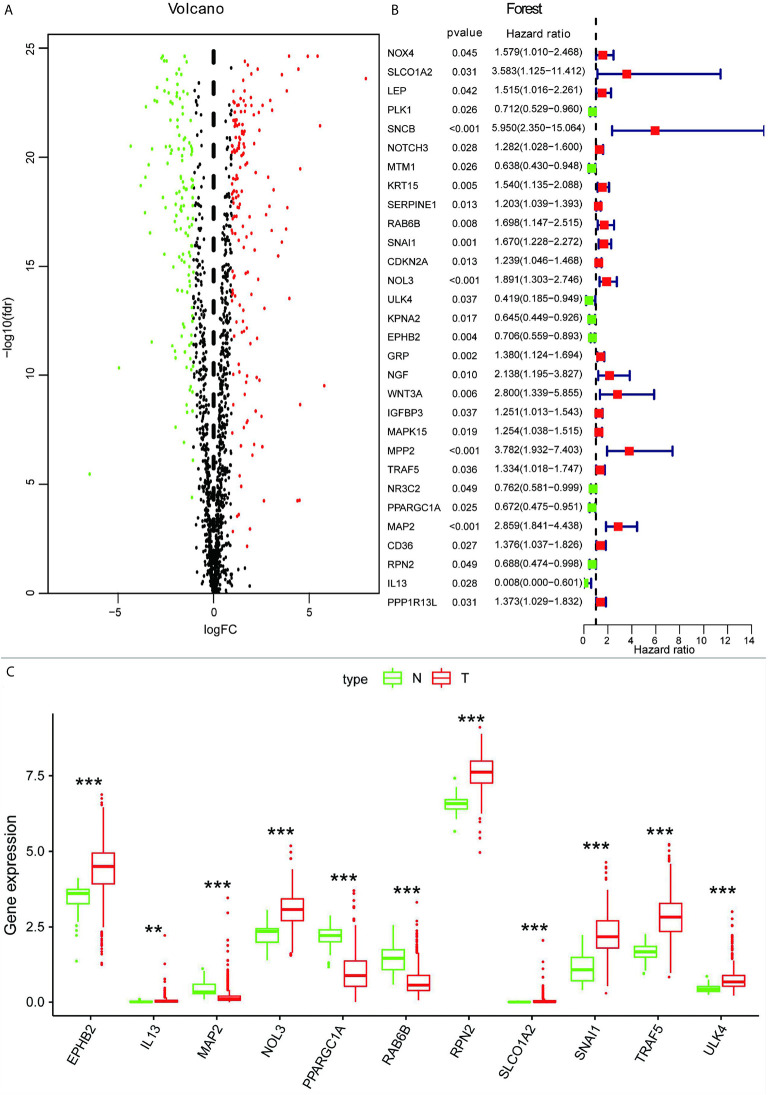
**(A)** Volcano map of autophagy-associated DEGs. **(B)** Screening of autophagy-associated genes associated with the prognosis of CRC by univariate Cox analysis. **(C)** Box plot showing expression of 11 autophagy-associated genes in normal tissues and CRC. **p < 0.01, ***p < 0.001.

According to the mRNA matrix data for 568 CRC tissues and 44 normal tissues in TCGA, absolute values of mRNA expression levels with log (fold change) >1.0 and FDR <0.05 were used as screening criteria. A total of 301 autophagy-associated DEGs were obtained, of which 134 were significantly downregulated and 167 were significantly upregulated. The scatter plot is shown in **Figure 1A**, and the top five autophagy genes with the most significant upregulation and downregulation are presented in **Table 1**.

**Table 1:** Top five autophagy-associated genes significantly upregulated and downregulated in CRC.

Gene	Full name	logFC	P value	FDR
*COL10A1*	Collagen Type X Alpha 1 Chain	8.019405808	3.63E-26	2.50E-24
*AQP5*	Aquaporin 5	5.844415496	1.27E-10	3.06E-10
*CLDN2*	Claudin 2	5.622786050	2.19E-23	3.62E-22
*INHBA*	Inhibin Subunit Beta A	5.494599523	5.10E-28	2.34E-25
*CLDN1*	Claudin 1	4.979004650	7.39E-28	2.38E-25
*HSPB8*	Heat Shock Protein Family B (Small) Member 8	-3.538252884	5.48E-19	3.46E-18
*NR1H4*	Nuclear Receptor Subfamily 1 Group H Member 4	-3.757236985	2.43E-20	1.98E-19
*MYH11*	Myosin Heavy Chain 11	-4.283574351	2.55E-22	3.10E-21
*CYP3A4*	Cytochrome P450 Family 3 Subfamily A Member 4	-4.896328336	1.75E-11	4.63E-11
*APOB*	Apolipoprotein B	-6.433852158	1.98E-06	3.41E-06

### Prognostic Model Construction

To further understand whether autophagy-associated DEGs were associated with the survival of CRC patients, we obtained 30 prognosis-associated genes by univariate Cox analysis (**Figure 1B**), which were screened and modeled by further multivariate Cox analysis (**Table 2**). Finally, a box plot was used to show the expression of genes used to build the model in normal and CRC tissues (**Figure 1C**).

**Table 2:** Construction of 11 autophagy-associated genes for prognosis.

No.	Gene	Full name	Coef	HR	Relevance score
1	*SLCO1A2*	Solute Carrier Organic Anion Transporter Family Member 1A2	1.24168405	3.461437794	2.75
2	*RAB6B*	Member RAS Oncogene Family	0.425074259	1.52970401	1.8
3	*SNAI1*	Snail Family Transcriptional Repressor 1	0.480196885	1.616392615	2.12
4	*NOL3*	Nucleolar Protein 3	0.47383047	1.606134675	1.94
5	*ULK4*	Unc-51 Like Kinase 4	-1.040144239	0.353403704	2.42
6	*EPHB2*	EPH Receptor B2	-0.295212905	0.74437309	2.18
7	*TRAF5*	TNF Receptor Associated Factor 5	0.379124034	1.461004239	1.94
8	*PPARGC1A*	PPARG Coactivator 1 Alpha	-0.516127562	0.596827255	2.84
9	*MAP2*	Microtubule Associated Protein 2	0.742235004	2.100625177	2
10	*RPN2*	Ribophorin II	-0.557629665	0.572564627	1.59
11	*IL13*	Interleukin 13	-6.473435484	0.001543913	3.19

### Assessing the Accuracy of the Prognostic Model

**Figure 2:**
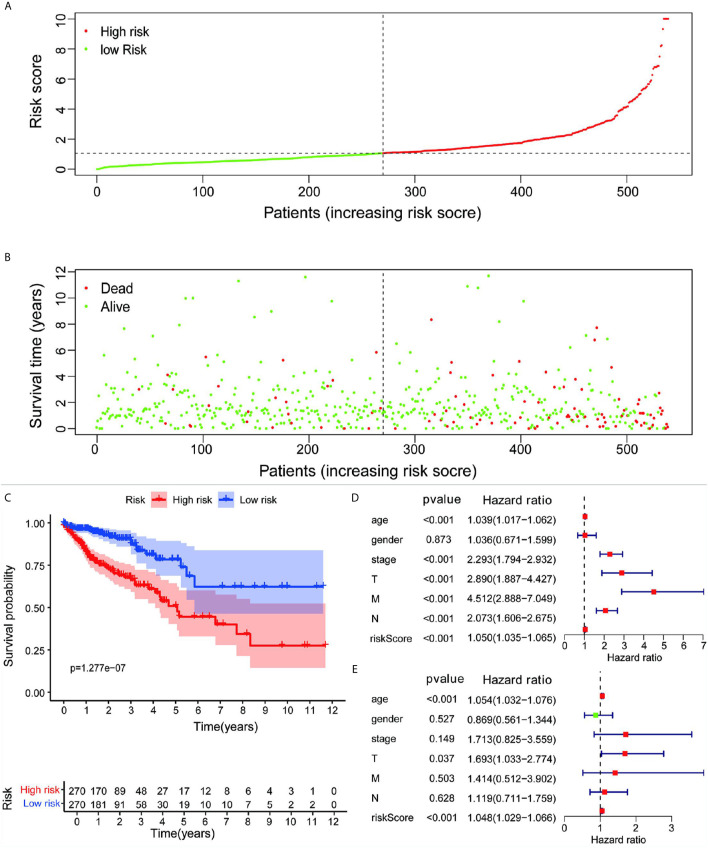
**(A)** Risk scores for all samples from clinical data. **(B)** Distribution of risk scores and survival time of patients. **(C)** KM assessment of survival time for samples from TCGA clinical data using the autophagy prognostic model. Forest plot of **(D)** univariate and **(E)** multivariate independent prognostic analysis of the model.

**Figure 3:**
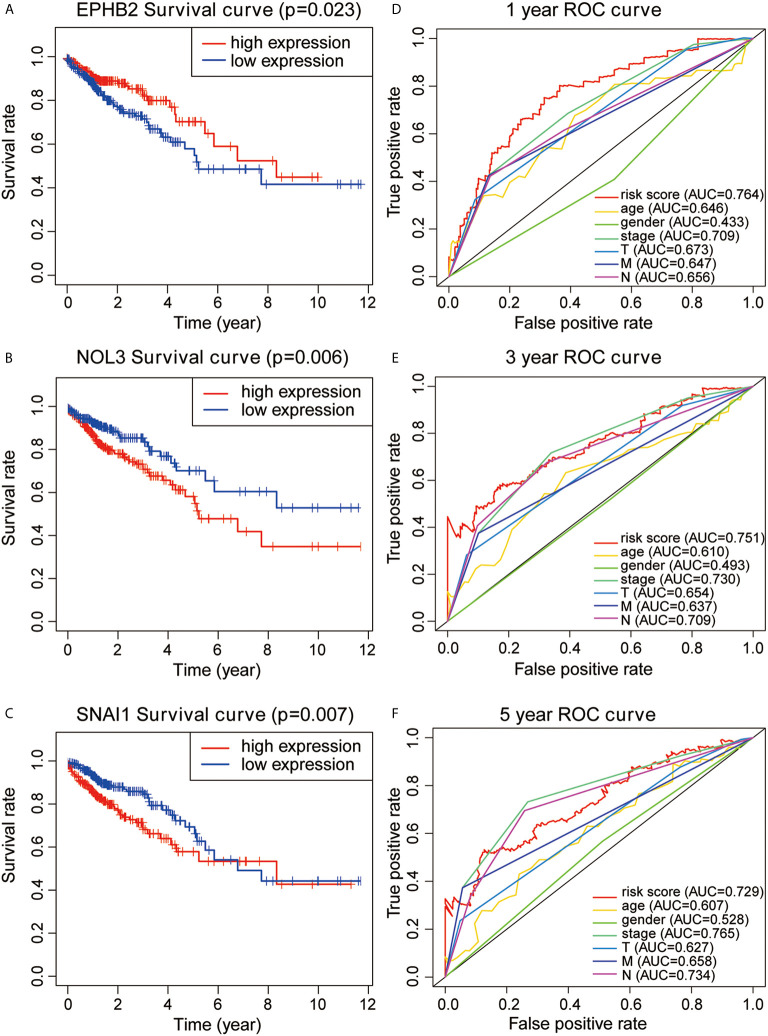
**(A–C)** KM survival analysis for EPHB2, NOL3, and SNAL1. **(D–F)** 1-year, 3-year, and 5-year ROC curves and AUC based on the autophagy prognostic model.

Based on the analysis results, the median value of the risk score was determined. According to the median value, all samples were classified into high- and low-risk groups. The analysis results were visualized and a distribution map of the risk score was drawn. **Figure 2A** shows the probability distribution of the risk score. Red points represent samples of the high-risk group, and green points represent samples of the low-risk group. **Figure 2B** presents the distribution of risk scores and survival time. The ordinate is the survival time (in years), the red points denote dead cases, and the green points denote alive cases. Survival analysis of risk scores showed significant statistical differences between the two groups (p = 1.277e-07) (**Figure 2C**). The results of KM survival analysis of the 11 autophagy prognostic genes in the model indicated that EPHB2, NOL3, and SNAI1 were relevant to the survival of patients with CRC (**Figures 3A–C**). According to the prognostic model, the survival rates of the high- and low-risk groups were predicted (**Table S1**). The 5-year survival rate of CRC patients in the low-risk group was approximately 0.738, with a 95% confidence interval (CI) of 0.618-0.881. The 5-year survival rate of CRC patients in the high-risk group was about 0.477 with a 95% CI of 0.365-0.623. Univariate and multivariate independent prognostic analyses uncovered that the risk score for this prognostic model was an independent prognostic factor (p < 0.001) (**Figures 2D, E**). The AUC values for the 1-year, 3-year, and 5-year ROC curves were 0.764, 0.751, and 0.729, respectively (**Figures 3D–F**). Finally, the C-index was applied to evaluate the prediction ability of the model. The C-index is the proportion of all patient pairs in which the predicted consequences are in agreement with the practical consequences. The C-index of the prognostic model was calculated to be 0.734 (standard error = 0.028). In summary, the above results show that the model has a moderate accuracy.

### Evaluation of EPHB2, NOL3, and SNAI1

**Figure 4:**
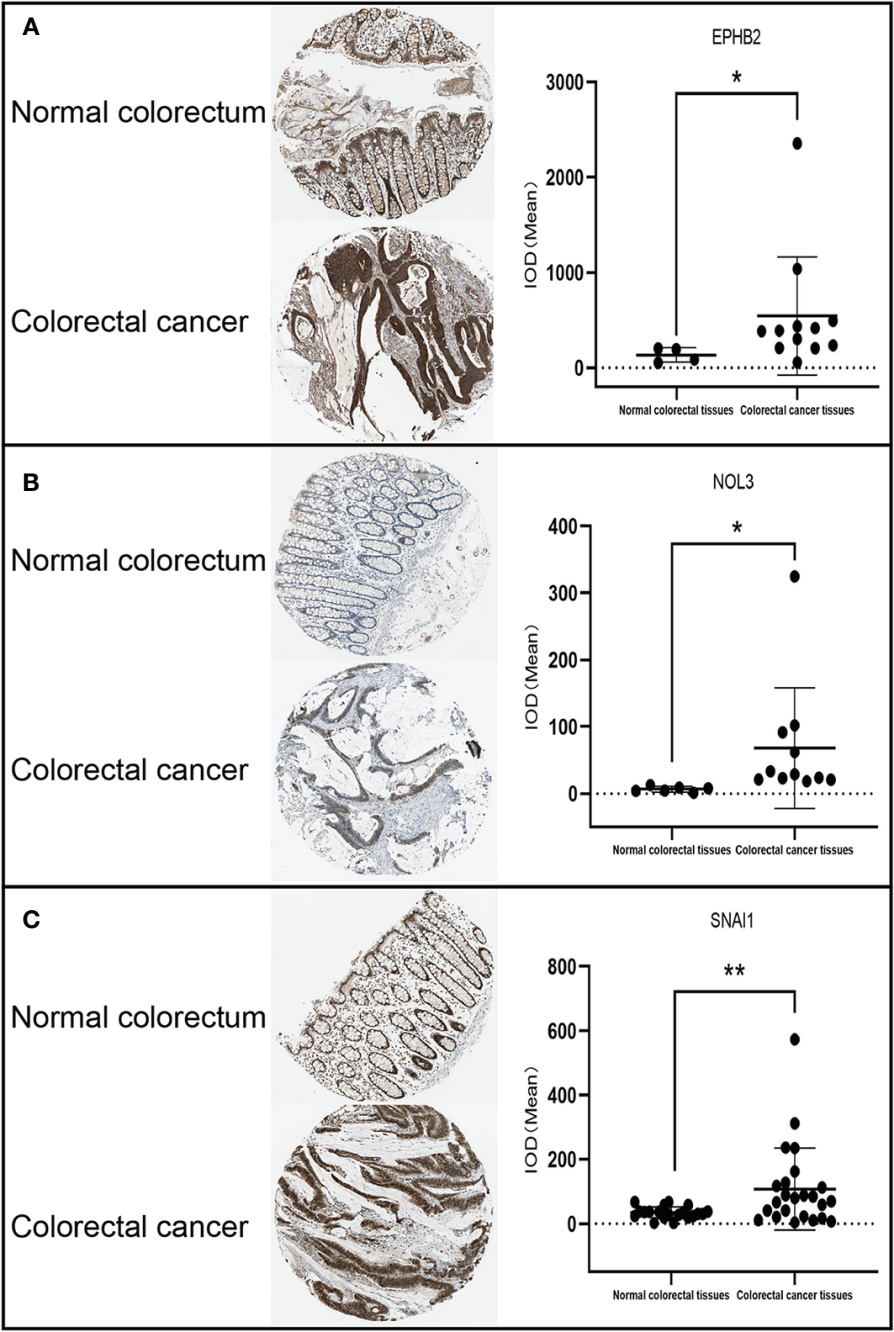
IHC analysis of EPHB2 **(A)**, NOL3 **(B)**, and SNAI1 **(C)** in CRC tissues. *p < 0.05, **p < 0.01.

**Figure 5:**
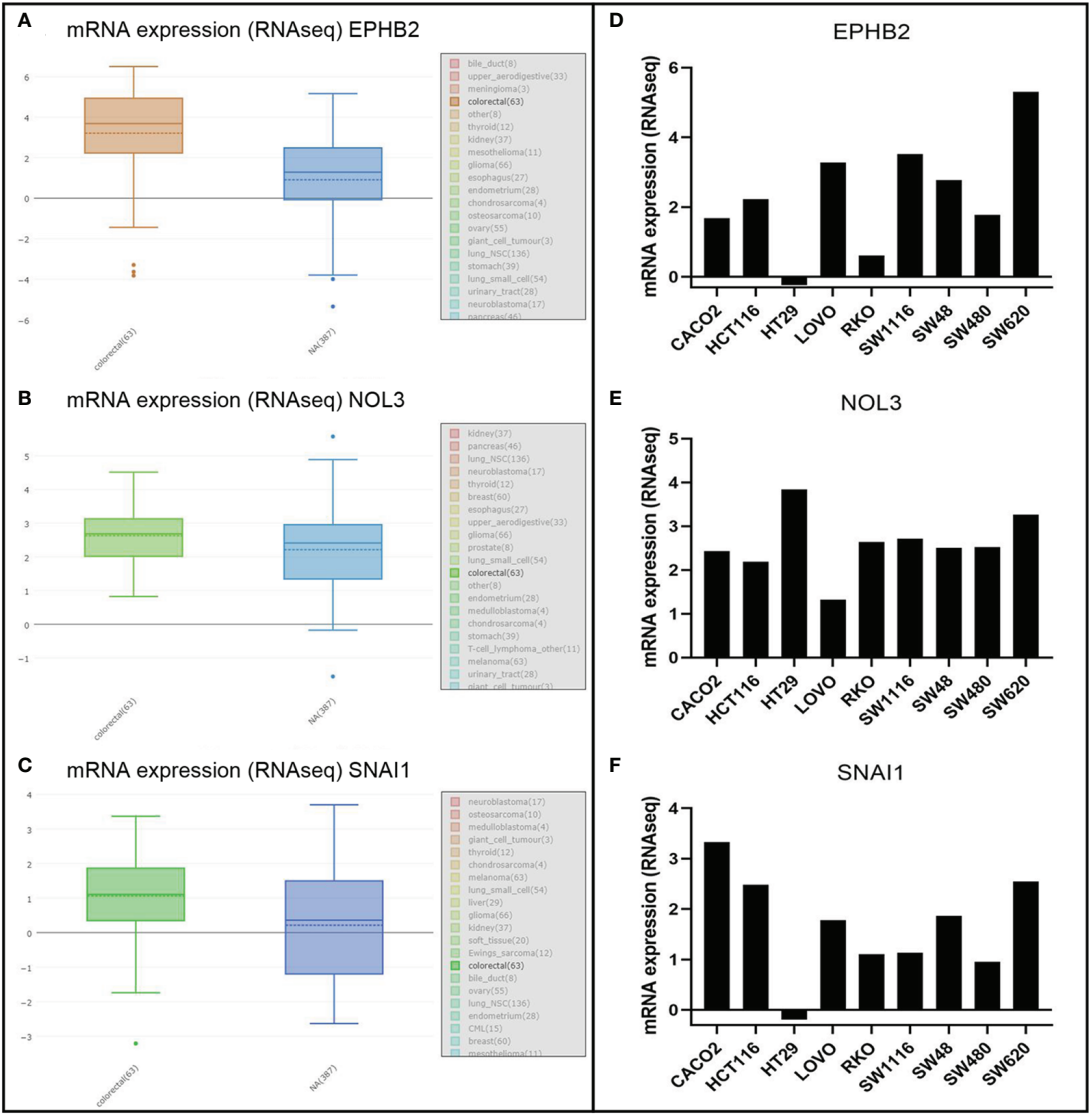
mRNA analysis of EPHB2 **(A, D)**, NOL3 **(B, E)**, and SNAI1 **(C, F)** in CRC cell lines.

EPHB2, NOL3, and SNAI1 were highly expressed at the protein level (**Figure 4**) and cellular level (**Figures 5A–C**), which were consistent with the data obtained in TCGA. The most upregulated expressions of EPHB2, NOL3, and SNAI1 in the nine common CRC cell lines were SW620, HT29, and CACO2, respectively (**Figures 5D–F**).

### Acquisition of Potential Signaling Pathways

**Figure 6:**
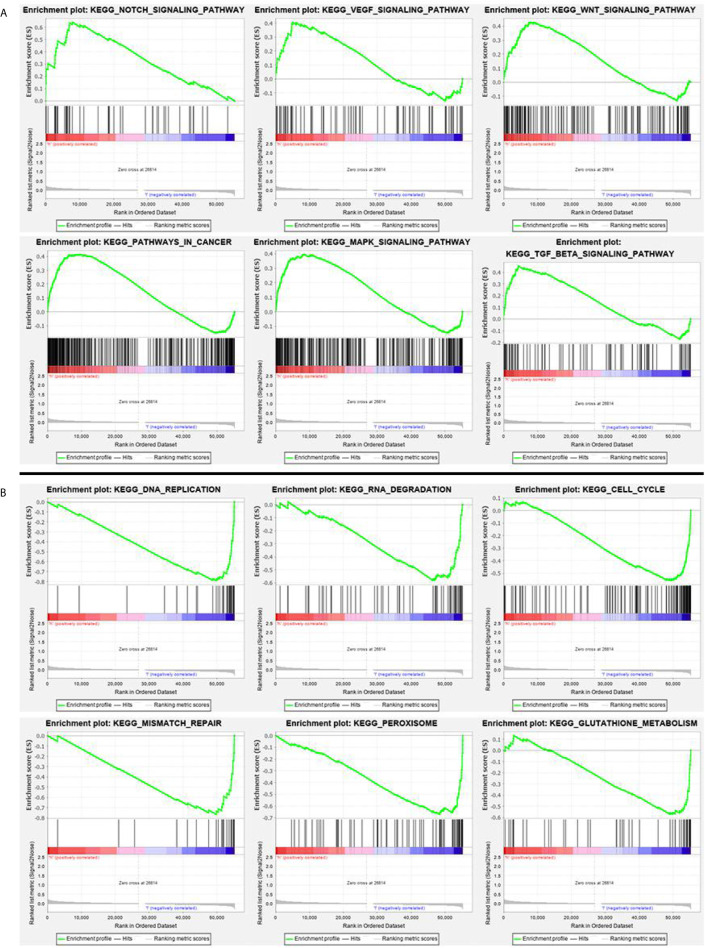
**(A)** GSEA analysis of main enriched pathways in the high-risk group. **(B)** GSEA analysis of main enriched pathways in the low-risk group.

**Figure 7:**
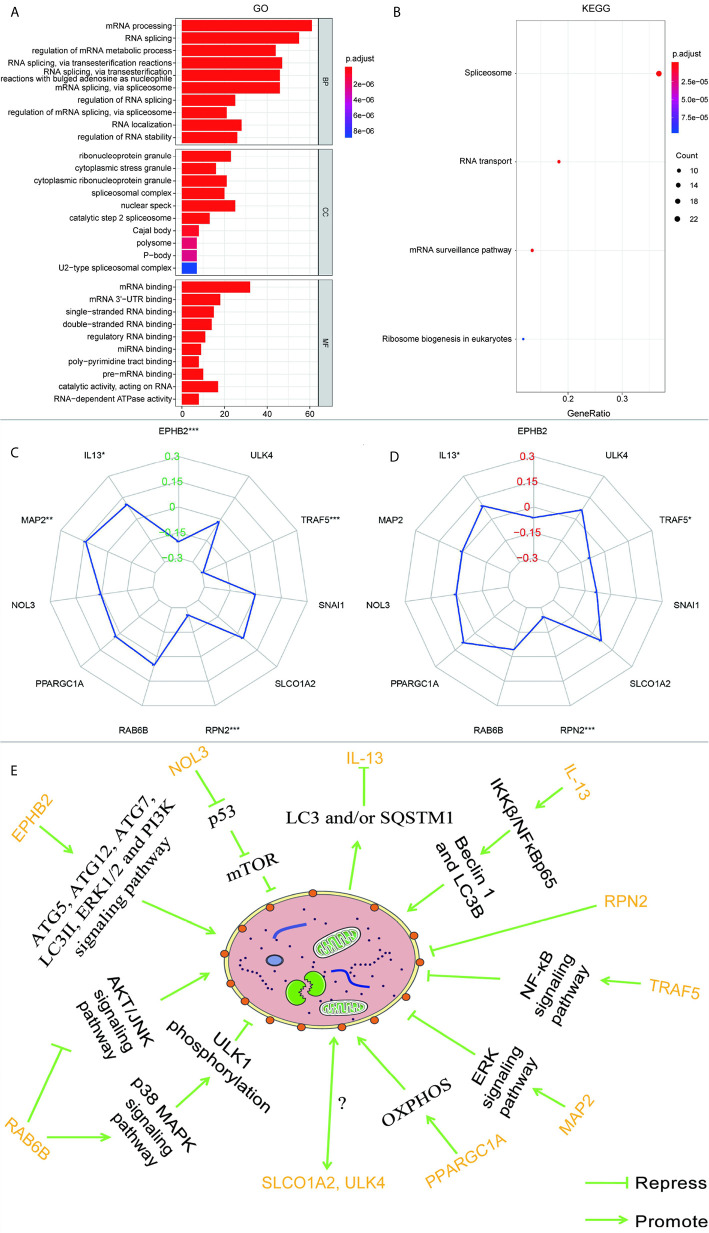
**(A)** GO analysis and **(B)** KEGG analysis of the target genes of 11 autophagy-associated genes in the model. The radar maps of 11 autophagy-associated genes are analyzed with MSI **(C)** and TMB **(D)**, respectively. Regulatory relationships between the 11 genes in the model and autophagy **(E)**. *p < 0.05, **p < 0.01, ***p < 0.001.

According to the GSEA analysis, the high-risk group was mainly associated with the Notch, VEGF, WNT, MAPK, and TGF-β signaling pathways, which are tumor-related pathways (**Figure 6A**; **Table 3**). The low-risk group had a negative correlation with DNA replication, RNA degradation, cell cycle, mismatch repair (MMR), peroxisome, and glutathione metabolism, which are mainly involved in cytoprotective response (**Figure 6B**; **Table 3**). GO analysis revealed that the primary biological processes of the target genes of the model included mRNA processing, RNA splicing, and regulation of mRNA metabolic process (**Figure 7A**). KEGG analysis revealed that the major enriched pathways included spliceosome, RNA transport, mRNA surveillance pathway, and ribosome biogenesis in eukaryotes (**Figure 7B**). These pathways are mainly concentrated in the processes of splicing and metabolism of mRNA.

**Table 3:** Display of GSEA enrichment results in high and low risk groups.

Name	^1^NES	^2^NOM *p*-val	^3^FDR *q*-val
Notch signaling pathway	2.1448512	0	0.006673426
Wnt signaling pathway	1.8196449	0.003795066	0.056357715
Pathways in cancer	1.7476273	0.021442495	0.07196092
TGF-β signaling pathway	1.7448014	0.021484375	0.06931268
MAPK signaling pathway	1.7017577	0.021526419	0.08378335
VEGF signaling pathway	1.6328211	0.02330097	0.11057795
DNA replication	-2.026768	0	0.021217022
RNA degradation	-1.93148	0.006147541	0.026900437
Cell cycle	-1.9373425	0.020283977	0.027462965
Mismatch repair	-2.0285935	0.004040404	0.024248024
Peroxisome	-2.2689974	0	0.011890192
Glutathione metabolism	-1.9067883	0.004065041	0.03125281

^1^NES represents the normalized enrichment score ^2^NOM p-val is the p value, which represents the credibility of the enrichment results ^3^FDR q-val represents the p value after correction by multiple hypothesis tests.

### Some Autophagy-Associated Genes Were Related to MSI and TMB

Based on the previous data, a correlation between MMR pathway and low-risk groups was discovered, so we speculate that the expression of these autophagy-associated genes may be related to MSI. A previous report has shown that high TMB in CRC is usually related to MSI and mismatch repair defects (19). Therefore, we also analyzed the relationship between these autophagy-related genes and TMB. As can be seen from **Figures 7C, D**, EPHB2, IL-13, MAP2, RPN2, and TRAF5 are correlated with MSI, EPHB2 (R = -0.204), RPN2 (R = -0.255), and TRAF5 (R = -0.295) are negatively correlated with MSI, while IL-13 (R = 0.102) and MAP2 (R = 0.141) are positively correlated. IL-13, RPN2, and TRAF5 were correlated with TMB, RPN2 (R = -0.244) and TRAF5 (R = -0.094) were negatively correlated with TMB, while IL-13 (R = 0.093) was positively correlated with TMB.

## Discussion

CRC is caused by abnormal cell growth in the colon or rectum. Increasing numbers of studies confirm the importance of autophagy in CRC at various stages (20, 21). In the current work, we used bioinformatics analysis to identify autophagy-associated genes with statistically significant differences in expression in CRC.

As the early symptoms of CRC are not obvious, the prognosis of CRC patients is poor once CRC progresses (22). Hence, the identification of effective prognostic markers is important to guide assessment and treatment of CRC patients. Huang et al. constructed a prognostic model that has been confirmed by experiments to have a clear correlation with CRC, and the marker based on multi-RNA had a higher prognostic accuracy than TNM staging (23). Zhou et al. identified five autophagy genes to establish an early recurrence classifier. The report shows that autophagy score can be used to predict the postoperative survival rate of CRC (24). Qian and his colleagues constructed a competitive endogenous RNA (ceRNA)-ceRNA interaction network mediated by autophagy in CRC by integrating the systematic expression profiles of long non-coding RNA and mRNA (25). Some people even screened the autophagy-related non-coding RNA, for network construction or prognostic model construction based on the characteristics of autophagy genes (26). Most of the above studies used the human autophagy database to obtain a small number of autophagy-related genes, in order to identify biomarkers related to the prognosis of CRC. However, these studies ignored the potential key autophagy-related genes that have not been paid attention to, which is of great significance in mining the prognosis, occurrence, and development of CRC. As autophagy affects the progression and prognosis of CRC and may even lead to chemotherapy resistance, we established a prognostic model of CRC based on 1,511 autophagy-associated genes. It was a new simple prognostic model based on the listed genes (SLCO1A2, RAB6B, SNAI1, NOL3, ULK4, EPHB2, TRAF5, PPARGC1A, MAP2, RPN2, and IL-13) and established using univariate Cox analysis and multivariate Cox analysis. ULK4, EPHB2, PPARGC1A, RPN2, and IL-13 had hazard ratio (HR) values less than 1 and were thus good prognostic factors, whereas SLCO1A2, RAB6B, SNAI1, NOL3, TRAF5, and MAP2 with a HR greater than 1 were considered poor prognostic factors. The 5-year survival rates of CRC patients in the high-risk group and low-risk group were 73.8% and 47.7%, respectively. These results indicate that the survival rates of patients with high-risk scores were markedly lower than those of patients with low-risk scores. We deduced the following rule from the risk curve: the higher the risk score, the greater the number of deaths and the larger the proportion of death. Independent prognostic analysis revealed that the risk value obtained from the autophagy prognostic prediction model was an independent prognostic factor. Higher risk scores were correlated with poorer patient prognosis.

According to the KM survival analysis, the autophagy-associated genes EPHB2, NOL3, and SNAI1 in the prognostic model were statistically significant (p < 0.05). *EPHB2* encodes the receptor tyrosine kinase transmembrane glycoprotein family member EPHB2, which engages in lots of cellular processes including movement, division, and differentiation (27). EPHB2 functions in the gastrointestinal homeostasis and is an essential factor regulating the classification of mature epithelial cells (28). EPHB2 primarily generates in epithelial cells and is the highest level of EPH receptor in the ordinary intestine (29). Studies in human breast cancer have shown that the expression of EPHB2 can induce the increase of ATG5/12 and LC3II, thereby inducing autophagy (26). Based on experimental results, Kandouz and colleagues propose that EPHB2 may affect autophagy *via* the ERK1/2 and PI3K pathways (27, 30). Moreover, they observed LC3 accumulation and transformation from LC3I to LC3II in EPHB2-upregulated cells. Knock-out of the autophagy regulatory genes ATG5/7 can significantly reduce cellular death induced by EPHB2. Some researchers have found that reducing tyrosine phosphorylation of EPHA1 and EPHB2 induces autophagy in CRC cells (28). There are also reports that the expression of EPHB2 is reduced during the development of CRC tumors, and its high expression may inhibit the development of tumors and reduce the invasion of cancer cells (31). This is consistent with the relationship between the low expression of EPHB2 and the poor prognosis of CRC in our survival analysis. EPHB2 is a tumor suppressor that affects the progression of CRC by acting on autophagy (27).

On the contrary, according to the results of our survival analysis, high expression of NOL3 was connected to poor prognosis in CRC. NOL3 encodes an anti-apoptotic protein that is involved in pathways including apoptosis and autophagy, apoptosis regulation, and signal transduction (32). The caspase recruitment domain (CARD) of NOL3 can downregulate the activity of p53 *via* CARD–CARD interaction (32). p53 is a well-known tumor suppressor protein (33). Some research indicates that p53 may induce autophagy whether it is inhibited or activated, and current data suggest that p53 promotes cell autophagy by inhibiting the mechanistic target of rapamycin kinase (mTOR) (34). SNAI1 participates in the stimulation of epithelial-to-mesenchymal transition (EMT) and exerts a vital role in tumor drug resistance, cellular proliferation inhibition, survival, and movement (35). SNAI1 is a pivotal regulator of EMT and controls CRC invasion and proliferation (36). Recent research suggests that autophagy degrades SNAI1 in cancer cells *via* LC3 and/or (sequestosome 1) SQSTM1, thereby inhibiting tumor progression (30).

Furthermore, the other eight genes in the model are closely related to the tumor. Some studies suggest that RAB6B, one of the RAS oncogene family members, functions in retrograde transport at the Golgi complex level or in retrograde transport in nerve cells (37). Silencing RAB6B in gastric cancer inhibits the AKT/JNK signaling pathway, suppressing gastric cancer cell proliferation, and impels apoptosis by furthering the p38 MAPK pathway (38). Overexpression of Caveolin-1 has been shown to reduce paclitaxel resistance of osteosarcoma cells *via* weakening autophagy, and the AKT/JNK pathway is an effective regulator for autophagy (39, 40). Furthermore, p38 MAPK can inhibit autophagy and promote microglial inflammation *via* phosphorylating unc-51-like autophagy activating kinase 1 (ULK1) (41), and the stimulation of the p38 MAPK pathway *via* osteopontin can advocate malignant change in CRC, and suppress autophagy (42).

SLCO1A2 is the gene encoding organic anion-transporting polypeptide 1A2 (OATP1A2), which belongs to the organic anion transport polypeptide (OATP) subunit of the superfamily of drug transporters. OATP1A2 is mainly found in epithelial tissue and can affect the distribution of many drugs, xenobiotics, and endophytes (43). OATP1A2 dysfunction may damage the pharmacokinetics and traits of a drug, thereby affecting the effectiveness of the treatment, and it may also hinder the absorption of endogenous organisms to the target tissue (43). OATP1A2 is regulated by AMPK, which has an impact on its membrane target, internalization, reuse, and degradation processes (44). Some researchers found that OATP1A2 could not be detected in the large intestine of normal controls when conducting intestinal transport protein research, but in another study, OATP1A2 was found to be widely expressed in patients with CRC liver metastases (45, 46). Therefore, OATP1A2 could be an influential factor altering the effect of oral drug treatment in CRC. As shown in **Figure 2**, the expression level of the SLCO1A2 tumor group was higher than that of the normal group. The interaction between autophagy and drug transporters is reported to be related to drug resistance (47). For example, high expression of the transporter ATP binding cassette subfamily G member 2 can promote autophagy, and the activation of autophagy greatly increases the survival rate of cells (48). TRAF5 regulates the stimulation of the typical nuclear factor kappa B (NF-κB) pathway (49, 50). Most investigations have found that activation of the NF-κB pathway can inhibit autophagy (51).

RPN2 is tremendously overexpressed in CRC and promotes cell proliferation by regulating the glycosylation state of epidermal growth factor receptor (52, 53). RPN2 has been manifested to repress autophagy in liver cancer (54). IL-13, one of the autophagy genes used to construct the model, is an important T cell-derived cytokine that induces EMT in CRC cells (55). In the airway epithelium, IL-13 can activate autophagy and affect cell secretion (56). Besides, in breast carcinoma, IL-13 can regulate the expression of Beclin 1 as well as light chain 3 beta (LC3B), increasing the formation of autophagosomes *via* IKKβ/NFκBp65 (57). The protein encoded by PPARGC1A is a transcriptional co-activator that modulates genes involved in energetic metabolism (58). This protein cooperates with a variety of transcription elements to enhance mitochondrial oxidative phosphorylation (OXPHOS) under conditions of high energy demand (58). In various malignant tumors, including CRC, ascendant expression of PPARGC1A is strongly related to metabolism and advances the growth, distant spread, and chemical resistance of tumor cells (58). Overexpression of PPARGC1A increases the amount of the OXPHOS protein complex, accelerates autophagy, and activates tumor development in breast cancer cells (59). In melanoma cells, downregulating PPARGC1A/PPARGC1B results in decreased OXPHOS activity, creating an acidic tumor environment and triggering autophagy (60). MAP2 is mainly involved in neurite outgrowth and neuronal migration during neuronal development (61, 62). However, there have been few studies of MAP2 in non-nerve cells (61). Some researchers have observed that a high level of MAP2 is absent from normal mucosa in aggressive oral neoplasm (61). MAP2 interacts with growth factor receptor-bound protein 2 to enhance the ERK signaling pathway (63). In RAS-driven cancers (including CRC), it has been demonstrated that RAF/MEK/ERK inhibition can cause cancer-cell-protective autophagy (63). ULK4 belongs to a member of the unc-51-like serine/threonine kinase family functioning in the neuron (64). Lebovitz et al. investigated more than 200 human autophagy-related signatures and cancer-associated changes in the DNA sequence as well as RNA expression, and used sequence data from TCGA to examine their relationships with multiple cancer types and patient survival outcomes (65). ATG7 and ULK4, which are core autophagy genes, showed effective selection of mutations in renal cancer and endometrial cancer, respectively, indicating that the expression and mutation of ULK4 may be closely related to autophagy (65). The above data demonstrate that the 11 genes used to build the model are all involved in autophagy to a greater or lesser extent, as illustrated in **Figure 7E**. However, owing to a lack of research, the mechanisms by which ULK4 and SLCO1A2 interact with autophagy remain unclear. They appear to be key genes regulating the autophagy pathway in CRC; however, more experiments are needed to confirm this.

Some signaling transduction pathways are known to be abnormally activated during the occurrence and progression of CRC, in particular, Notch, VEGF, WNT, MAPK, and TGF-β signaling, and these pathways cooperate with cell autophagy to determine the fate of the cell (66–69). Our GSEA enrichment analysis showed that the high-risk group with higher mortality than the low-risk group had a mainly positive role in the relevant mechanisms of tumor cells, including the Notch, VEGF, WNT, MAPK, and TGF-β signaling pathways, 11 autophagy associated genes may be related to the biological pathway related to CRC, and its dysfunction may lead to poor prognosis of CRC. While the low-risk group had mainly negative correlations with DNA replication, RNA degradation, cell cycle, MMR, peroxisome, and glutathione metabolism, all of which are normal cell protective responses. A considerable number of studies have concluded that autophagy is a protective mechanism for cancer cells, limiting the response to various therapeutic interventions (70, 71). These results indicate that autophagy is largely based on the interaction with various abnormal signaling pathways to influence the progress of CRC. A large number of studies have proved that the MMR pathway plays an important role in repairing DNA replication errors in normal and tumor cells (72, 73). The deficiency of DNA MMR protein, which determines the microsatellite instability-high (MSI-H), may lead to the accumulation of mutations and the production of new antigens, which may stimulate the anti-tumor immune response (74). Clinical trials have shown that MSI-H status is associated with long-term benefits for patients treated with an immune checkpoint inhibitor. It has been reported that the expression level of autophagy key factor LC3B-II in CRC is higher than that in MSI (75). UVRAG, a key autophagy tumor suppressor, produces truncated mutations when CRC patients have MSI (76). The mutated UVRAG loses its ability to inhibit autophagy and promotes tumorigenesis (76). A recent study report also suggested that autophagy-related genes were expressed differently in an MSI group and microsatellite stability (MSS) group, which suggested that autophagy may be closely related to tumor MSS. According to our analysis of the results, we found that EPHB2, IL-13, MAP2, RPN2, and TRAF5 are related to MSI, and EPHB2 has been experimentally confirmed to be negatively related to MSI-H (77), which is the same as the result of our analysis. From the results, we discovered that RPN2 and TRAF were negatively correlated with TMB and MSI, while IL-13 was positively correlated with TMB and MSI. The correlation between RPN2, TRAF, and IL-13 in TMB and MSI was consistent, which partly explains the consistency with high TMB and high MSI, and suggests that autophagy-related genes may have a key effect on MSI.

At the same time, to further understand the potential mechanism between the 11 autophagy-associated genes of this model and CRC, we analyzed their targeted genes by GO and KEGG. According to **Figure 7B**, all the targeting genes of the 11 autophagy-associated genes used to construct the prognostic model may be related to RNA splicing. Splicing factors are key regulators of the mRNA alternative splicing (78). Researchers have investigated the expression level of more than 20 splicing factors, meanwhile autophagy was stimulated by hypoxia in oral tumors and found that the serine and arginine rich splicing factor 3 (SRSF3) was considerably downregulated (78). The results show that the splicing factor SRSF3 is a carcinogen in CRC, and the silencing of its expression can induce autophagic death of CRC cells (79). The variable transcripts of Beclin 1 are produced by selective 3’ splicing, and its translation products show reduced activity during starvation-induced autophagy, suggesting that the subtype of splicing may be a negative regulator of autophagy (80). As a proliferation regulatory factor of CRC, splicing factor proline and glutamine rich (SFPQ) can cooperate with peroxisome proliferator activated receptor gamma (PPARγ) (81). PSF gene knockout induces autophagosome generation *via* suppressing PPARγ (81). In malignant tumor cells, knockdown of the core spliceosome components small nuclear ribonucleoprotein polypeptide E and small nuclear ribonucleoprotein D1 polypeptide resulted in cancer cell death through autophagy rather than apoptosis (82). Consequently, preceding investigations also support our hypothesis that RNA splicing affects the progression of CRC by regulating autophagy. However, the connection between the conservative processes (autophagy and RNA splicing) is not completely clear, and more experiments are needed.

The series of bioinformatics analyses described here show that the autophagy prognostic model had a certain level of accuracy in predicting the prognosis of CRC patients. Nevertheless, more research and clinical evidence are needed to confirm the validity of this model.

In total, 301 DEGs in CRC were identified by bioinformatics analysis. GSEA, GO, and KEGG analyses indicated that autophagy may have an essential function in key signaling pathways of CRC, thereby deepening our understanding of the mechanism by which autophagy participates in the development of CRC. The analysis of the relationship between autophagy-associated genes and MSI and TMB suggests that autophagy may be an important process affecting MSI and TMB. We also established a simple prognostic model using bioinformatics tools. The model was based on screening autophagy-associated genes that were extremely relevant to the appearance and evolvement of cancers. A series of analysis methods were used to assess the predictive capability of the model. We believe that the autophagy prognostic model might have a far-reaching role in improving traditional TNM staging and histological classification for the prognosis prediction and treatment of CRC patients.

## Data Availability Statement

Publicly available datasets were analyzed in this study. These can be found in The Cancer Genome Atlas (https://portal.gdc.cancer.gov/).

## Author Contributions

QH conducted data analysis and drafted manuscripts. ZL, JY, YL, and XL were involved in research design and data collection. Finally, WZ revised the manuscript. All authors contributed to the article and approved the submitted version.

## Funding

This work was supported by the National Natural Science Foundation of China (81472275, 81702399) and the Guangdong Basic and Applied Basic Research Foundation (2020A151501303, 2014A030313542, 2017A030313618).

## Conflict of Interest

The authors declare that the research was conducted in the absence of any commercial or financial relationships that could be construed as a potential conflict of interest.
