# Supplementary material for: Prognostic Significance of Autophagy-Relevant Gene Markers in Colorectal Cancer
Source: Front Oncol. 2021 Apr 15;11:566539. doi: 10.3389/fonc.2021.566539 (PMC8081889; doi:10.3389/fonc.2021.566539)
Supplement: Supplementary file 1 [file Table_1.doc]

**Table S1** According to the autophagy prognostic model, the survival rates of the low-risk groups and the high-risk groups are predicted.

| risk=high | | | | | |
| --- | --- | --- | --- | --- | --- |
| Time (year) | n .risk | n. event | survival | lower 95% CI | upper 95% CI |
| 0.000 | 270 | 5 | 0.981 | 0.966 | 0.998 |
| 0.118 | 244 | 1 | 0.977 | 0.960 | 0.995 |
| 0.134 | 242 | 1 | 0.973 | 0.954 | 0.993 |
| 0.162 | 241 | 2 | 0.965 | 0.943 | 0.988 |
| 0.167 | 239 | 3 | 0.953 | 0.928 | 0.979 |
| 0.247 | 233 | 1 | 0.949 | 0.923 | 0.976 |
| 0.266 | 230 | 1 | 0.945 | 0.917 | 0.973 |
| 0.332 | 229 | 1 | 0.941 | 0.912 | 0.970 |
| 0.334 | 228 | 1 | 0.937 | 0.907 | 0.967 |
| 0.419 | 224 | 2 | 0.928 | 0.897 | 0.961 |
| 0.427 | 222 | 1 | 0.924 | 0.892 | 0.958 |
| 0.436 | 221 | 1 | 0.920 | 0.887 | 0.954 |
| 0.471 | 219 | 1 | 0.916 | 0.882 | 0.951 |
| 0.482 | 218 | 1 | 0.912 | 0.877 | 0.948 |
| 0.490 | 217 | 1 | 0.907 | 0.872 | 0.944 |
| 0.564 | 212 | 1 | 0.903 | 0.867 | 0.941 |
| 0.586 | 210 | 1 | 0.899 | 0.862 | 0.937 |
| 0.625 | 208 | 1 | 0.895 | 0.857 | 0.934 |
| 0.647 | 207 | 1 | 0.890 | 0.852 | 0.930 |
| 0.663 | 206 | 1 | 0.886 | 0.847 | 0.927 |
| 0.718 | 200 | 1 | 0.881 | 0.842 | 0.923 |
| 0.795 | 196 | 1 | 0.877 | 0.837 | 0.919 |
| 0.827 | 194 | 1 | 0.872 | 0.831 | 0.916 |
| 0.838 | 191 | 3 | 0.859 | 0.816 | 0.904 |
| 0.907 | 184 | 1 | 0.854 | 0.810 | 0.900 |
| 0.915 | 182 | 1 | 0.849 | 0.805 | 0.896 |
| 0.918 | 181 | 1 | 0.845 | 0.800 | 0.892 |
| 0.926 | 178 | 1 | 0.840 | 0.794 | 0.888 |
| 0.978 | 174 | 1 | 0.835 | 0.789 | 0.884 |
| 0.989 | 173 | 1 | 0.830 | 0.783 | 0.880 |
| 1.008 | 169 | 1 | 0.825 | 0.778 | 0.876 |
| 1.049 | 164 | 1 | 0.820 | 0.772 | 0.871 |
| 1.085 | 159 | 2 | 0.810 | 0.761 | 0.863 |
| 1.104 | 156 | 1 | 0.805 | 0.755 | 0.858 |
| 1.162 | 147 | 1 | 0.799 | 0.749 | 0.854 |
| 1.167 | 142 | 1 | 0.794 | 0.742 | 0.849 |
| 1.170 | 138 | 1 | 0.788 | 0.736 | 0.844 |
| 1.353 | 121 | 1 | 0.781 | 0.728 | 0.838 |
| 1.359 | 120 | 1 | 0.775 | 0.721 | 0.833 |
| 1.400 | 118 | 1 | 0.768 | 0.713 | 0.828 |
| 1.523 | 105 | 1 | 0.761 | 0.705 | 0.822 |
| 1.586 | 104 | 1 | 0.754 | 0.697 | 0.816 |
| 1.836 | 93 | 1 | 0.746 | 0.687 | 0.809 |
| 1.868 | 91 | 1 | 0.737 | 0.678 | 0.802 |
| 2.000 | 89 | 1 | 0.729 | 0.668 | 0.796 |
| 2.077 | 83 | 1 | 0.720 | 0.658 | 0.789 |
| 2.205 | 76 | 1 | 0.711 | 0.647 | 0.781 |
| 2.252 | 73 | 1 | 0.701 | 0.636 | 0.773 |
| 2.463 | 67 | 1 | 0.691 | 0.624 | 0.765 |
| 2.718 | 59 | 1 | 0.679 | 0.610 | 0.756 |
| 2.997 | 49 | 1 | 0.665 | 0.593 | 0.746 |
| 3.173 | 39 | 1 | 0.648 | 0.571 | 0.735 |
| 3.184 | 38 | 1 | 0.631 | 0.551 | 0.723 |
| 3.784 | 31 | 1 | 0.611 | 0.525 | 0.710 |
| 4.090 | 27 | 1 | 0.588 | 0.497 | 0.695 |
| 4.290 | 24 | 1 | 0.564 | 0.467 | 0.680 |
| 4.332 | 23 | 1 | 0.539 | 0.438 | 0.663 |
| 4.688 | 18 | 1 | 0.509 | 0.402 | 0.644 |
| 5.066 | 16 | 1 | 0.477 | 0.365 | 0.623 |
| 5.153 | 15 | 1 | 0.445 | 0.330 | 0.601 |
| 6.781 | 10 | 1 | 0.401 | 0.279 | 0.577 |
| 7.729 | 7 | 1 | 0.344 | 0.214 | 0.551 |
| 8.334 | 5 | 1 | 0.275 | 0.144 | 0.524 |
| risk=low | | | | | |
| Time (year) | n .risk | n. event | survival | lower 95% CI | upper 95% CI |
| 0.00274 | 260 | 2 | 0.992 | 0.982 | 1.000 |
| 0.07945 | 256 | 1 | 0.988 | 0.976 | 1.000 |
| 0.08219 | 255 | 1 | 0.985 | 0.970 | 1.000 |
| 0.16712 | 243 | 1 | 0.981 | 0.964 | 0.998 |
| 0.24932 | 239 | 1 | 0.976 | 0.958 | 0.995 |
| 0.40000 | 232 | 1 | 0.972 | 0.952 | 0.993 |
| 0.41918 | 230 | 1 | 0.968 | 0.946 | 0.990 |
| 0.51507 | 221 | 1 | 0.964 | 0.940 | 0.987 |
| 1.10959 | 168 | 1 | 0.958 | 0.932 | 0.984 |
| 1.21096 | 159 | 1 | 0.952 | 0.924 | 0.980 |
| 1.29315 | 153 | 1 | 0.946 | 0.916 | 0.977 |
| 1.56164 | 116 | 1 | 0.937 | 0.904 | 0.972 |
| 1.76164 | 99 | 1 | 0.928 | 0.890 | 0.967 |
| 2.03562 | 90 | 1 | 0.918 | 0.876 | 0.962 |
| 2.35068 | 80 | 1 | 0.906 | 0.859 | 0.956 |
| 3.00000 | 58 | 1 | 0.891 | 0.836 | 0.949 |
| 3.00274 | 57 | 1 | 0.875 | 0.814 | 0.940 |
| 3.24658 | 46 | 1 | 0.856 | 0.787 | 0.931 |
| 3.25479 | 44 | 1 | 0.836 | 0.761 | 0.920 |
| 3.69315 | 36 | 1 | 0.813 | 0.728 | 0.908 |
| 4.11781 | 28 | 1 | 0.784 | 0.688 | 0.894 |
| 5.23288 | 17 | 1 | 0.738 | 0.618 | 0.881 |
| 5.48767 | 14 | 1 | 0.685 | 0.545 | 0.862 |
| 5.84658 | 11 | 1 | 0.623 | 0.464 | 0.837 |
